# The Roles of Post-Translational Modifications on mTOR Signaling

**DOI:** 10.3390/ijms22041784

**Published:** 2021-02-11

**Authors:** Shasha Yin, Liu Liu, Wenjian Gan

**Affiliations:** Department of Biochemistry and Molecular Biology, Medical University of South Carolina, Charleston, SC 29425, USA; yin@musc.edu (S.Y.); lliu@musc.edu (L.L.)

**Keywords:** mTOR, post-translational modifications, inhibitors, human diseases

## Abstract

The mechanistic target of rapamycin (mTOR) is a master regulator of cell growth, proliferation, and metabolism by integrating various environmental inputs including growth factors, nutrients, and energy, among others. mTOR signaling has been demonstrated to control almost all fundamental cellular processes, such as nucleotide, protein and lipid synthesis, autophagy, and apoptosis. Over the past fifteen years, mapping the network of the mTOR pathway has dramatically advanced our understanding of its upstream and downstream signaling. Dysregulation of the mTOR pathway is frequently associated with a variety of human diseases, such as cancers, metabolic diseases, and cardiovascular and neurodegenerative disorders. Besides genetic alterations, aberrancies in post-translational modifications (PTMs) of the mTOR components are the major causes of the aberrant mTOR signaling in a number of pathologies. In this review, we summarize current understanding of PTMs-mediated regulation of mTOR signaling, and also update the progress on targeting the mTOR pathway and PTM-related enzymes for treatment of human diseases.

## 1. Introduction

The mechanistic target of the rapamycin (mTOR) pathway is a central sensor of environmental clues, such as growth factors, nutrients, and energy, through which it controls proper cell growth and metabolism. Imbalances in the mTOR pathway including full inhibition or constitutive activation may disrupt the cellular homeostasis, leading to human diseases including but not limited to cancers, metabolic diseases, and aging and neurodegenerative disorders [1,2,3]. In the past three decades, studies have been exponentially expanded to map the landscape of the mTOR pathway, providing insightful knowledge for targeting this fundamental pathway to treat human diseases, particularly cancers.

**Figure 1:**
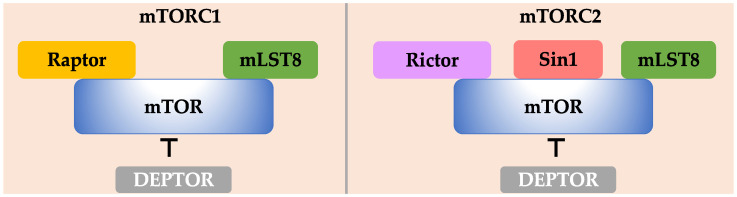
Core components of mTORC1 and mTORC2 complexes. mTOR and mLST8 are two shared subunits. Raptor defines the mTORC1 complex, while Rictor and Sin1 define mTORC2. DEPTOR is an endogenous inhibitor of both mTORC1 and mTORC2 complexes.

mTOR is an evolutionarily conversed protein kinase from yeast to human. In mammals, mTOR is the catalytic subunit of two different complexes (Figure 1), termed mTOR complex 1 (mTORC1) and mTOR complex 2 (mTORC2). These two complexes share two common subunits: mTOR and mLST8 (also known as GβL). The core component of mTORC1 includes Raptor, while mTORC2 contains Rictor and Sin1 (also known as MIP1 for MEKK2 interacting protein 1) [4,5,6,7]. Among these components, mLST8 is required for stabilization of the mTOR kinase domain [8]. Raptor is essential for mTORC1 cellular localization and substrate recruitment, as well as conferring mTORC1 rapamycin-sensitivity [9,10]. Sin1 is indispensable for the maintenance of mTORC2 integrity and substrates binding [11] and Rictor defines mTORC1 rapamycin insensitivity [7]. In addition, DEPTOR (DEP domain-containing mTOR-interacting protein) binds both mTORC1 and mTORC2 and functions as an endogenous inhibitor of both complexes [12].

mTORC1 exerts its biological functions by phosphorylating a large number of downstream proteins. mTORC1 phosphorylates S6K and 4E-BP1 to promote protein synthesis [13]. mTORC1 also promotes lipid synthesis by promoting Lipin 1 phosphorylation and consequent activation of the transcriptional programs driven by SREBP1/2 and PPARγ [14]. Moreover, mTORC1 was reported to stimulate de novo synthesis of purine and pyrimidine through ATF4/MTHFD2 and S6K1/CAD, respectively [15,16,17]. Furthermore, mTORC1 suppresses autophagy by directly phosphorylating ULK1, ATG13, UVRAG, and TFEB [18].

**Figure 2:**
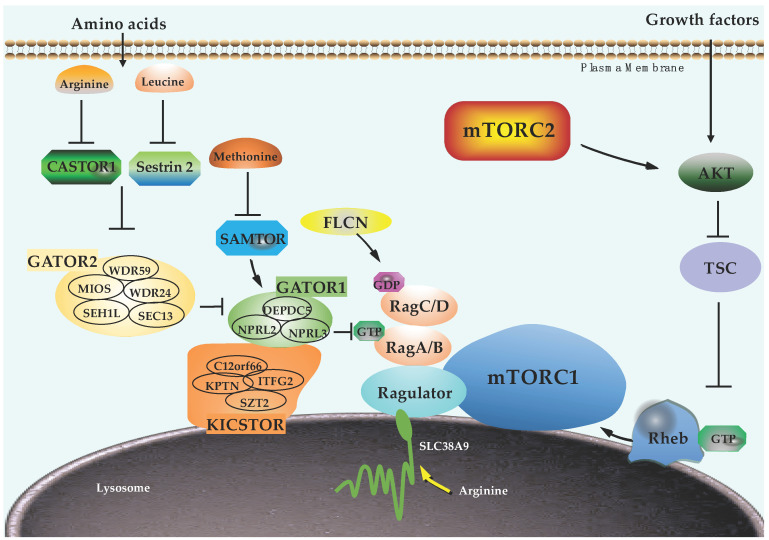
Amino acids and growth factors-mediated activation of the mTORC1 signaling. Amino acids bind to their specific sensors and transduce the signal through GATOR2, GATOR1, KICSTOR, and Ragulator complexes, leading to activation of Rag small GTPases and lysosomal recruitment of mTORC1. Growth factors activate other lysosomal localized small GTPases Rheb through Akt/TSC signaling axis. The small GTPases Rags and Rheb form two arms to fully activate mTORC1 pathway in response to amino acids and growth factors.

mTORC1 activation is spatially and temporally controlled by upstream inputs, notably amino acids and growth factors. In response to amino acids stimulation, Rag GTPases (Rags) are recruited to the lysosome by the Ragulator complex (p18, p14, MP1, C7orf59, and HBXIP) [19,20]. Subsequently, Ragulator functions as a guanine nucleotide exchange factor (GEF) to load GTP on RagA/B and the FLCN/FNIP complex acts as a GTPase-activating protein (GAP) to load GDP on RagC/D, promoting formation of the heterodimer consisting of GTP-loaded RagA/B and GDP-loaded RagC/D [19,21]. Activated Rags directly interact with Raptor to recruit mTORC1 to lysosome [22], where it binds to Rheb GTPase for maximal activation [23,24]. Unlike Rags, Rheb activity is primarily controlled by growth factors through the Akt/TSC signaling axis [25,26,27,28]. In addition to the abovementioned regulators, many proteins have also been identified to mediate the amino acids-induced activation of mTORC1 in the past fifteen years, including the GATOR1 complex (a GAP for RagA/B [29]), GATOR2 complex (an inhibitor of GATOR1 with unknown function [29]), KICSTOR complex (a mediator of GATOR1 lysosomal localization [30,31]), Sestrin2 (leucine sensor [32,33]), SAMTOR (S-adenosylmethionine sensor [34]), and SLC38A9 and CASTOR1 (arginine sensors [35,36]). Collectively, these components form a sophisticated network centering on the Rags and Rheb GTPases to regulate mTORC1 activation in response to amino acids and growth factors (Figure 2).

**Figure 3:**
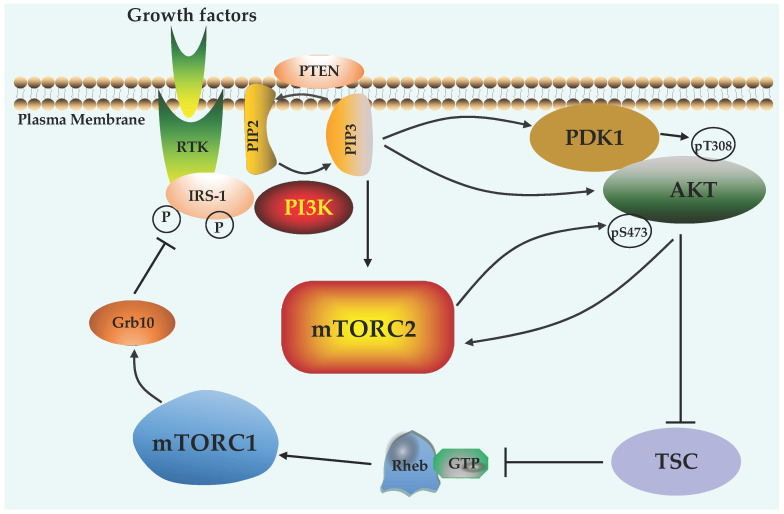
Crosstalk between mTOR signaling and PI3K/Akt pathway. Growth factors bind to receptor tyrosine kinase (RTK) and activate PI3K to convert PIP2 to PIP3, which can be reversed by PTEN. PIP3 recruits Akt to the plasma membrane for phosphorylation at T308 and S473 by PDK1 and mTORC2, respectively. Activated Akt suppresses TSC complex to promote GTP loading on Rheb for mTORC1 activation. In turn, mTORC1 phosphorylates Grb10 to inhibit RTK/PI3K activity. Thus, PI3K, Akt, and mTOR form a feedback regulatory loop.

Unlike mTORC1, mTORC2 is insensitive to rapamycin and has distinct roles on metabolism, cytoskeleton regulation, cell proliferation, and survival [7,11,37]. These biological functions of mTORC2 are achieved by phosphorylating a set of substrates including PKB/Akt, PKC, and SGK1 [38]. Of note, Akt functions as the key downstream effector of insulin signaling to connect mTORC2 with glucose homeostasis, adipogenesis, diabetes, and cancers [39]. Different from mTORC1, mTORC2 is primarily activated by growth factors in a PI3K-dependent manner (Figure 3). Specifically, in the absence of growth factors, the Sin1-PH domain interacts with the mTOR kinase domain, leading to mTOR inactivation. Upon growth factor stimulation, activated PI3K produces phosphatidylinositol (3,4,5)-trisphosphate (PIP3), which interacts with the Sin1-PH domain and releases its inhibitory effect, thereby triggering mTORC2 activation [40]. In addition, the mTORC2 activation is also regulated by small GTPases, including Rac1, Rap1, and Ras [41,42].

Having discovered dozens of proteins in the mTOR pathway, it is important to understand how these components are regulated, which may provide explanations for the aberrant mTOR signaling in human diseases. Besides genetic alterations and transcriptional regulations, post-translational modifications (PTMs) including phosphorylation, ubiquitination, acetylation, and glycosylation have been demonstrated to be key regulators of mTOR signaling. In this review, we will summarize PTMs-mediated regulation of the mTOR pathway (Table 1) and discuss strategies for targeting the mTOR pathway to treat human diseases.

## 2. Roles of PTMs on Shared Components of the mTOR Signaling

### 2.1. mTOR

Studies have demonstrated that mTOR kinase activity can be regulated by several types of PTM, notably phosphorylation. Growth factors promote the phosphorylation of mTOR at S2448 by Akt or S6K1 [43,44,45,46], which is considered as a marker of mTORC1 activation. Moreover, nutrients deprivation induces AMPK-dependent phosphorylation of mTOR at S2446 [47]. Interestingly, phosphorylation of these two residues antagonizes each other, providing a switch to control mTOR activity by nutrients and growth factors. Furthermore, PI3K/Akt/TSC/Rheb signaling-mediated mTOR phosphorylation at S1261 enhances mTORC1 kinase activity and cell growth [48]. mTOR also undergoes autophosphorylation at S2481, which is mTORC2-dependent and wortmannin-sensitive, but rapamycin-, amino acid-, and serum-insensitive [49,50]. However, Soliman et al. reported that growth factors promote mTOR-S2481 phosphorylation in both mTORC1 and mTORC2 complexes and rapamycin treatment or amino acid deprivation blocks mTORC1-associated S2481 phosphorylation [51]. Therefore, phosphorylation of mTOR-S2481 can be used as another marker of mTOR activation. In addition, dual phosphorylation of mTOR at S2159/T2164 weakens Raptor interaction with mTOR and PRAS40, leading to enhanced mTOR-S2481 autophosphorylation and mTORC1 intrinsic kinase activity [52]. Interestingly, levels of mTOR-pS2481 are significantly elevated in Alzheimer’s disease (AD) and positively correlated with tau phosphorylation [53], while phosphorylation of mTOR-S2448 is stronger in metastatic cancer than in primary cancer of liver and kidney [54]. Ubiquitination is also a critical PTM in regulating mTOR activation. The E3 ubiquitin ligase FBXW7 promotes mTOR ubiquitination and subsequent proteasome-mediated degradation, leading to termination of mTOR signaling [55,56]. Consistently, frequent loss of FBXW7 in breast cancers is associated with hyperactivation of mTORC1 and increases sensitivity to rapamycin [55]. Moreover, Parkin-catalyzed polyubiquitination at K2066 and K2306 is required for mTORC1 activation and cell survival under mitochondrial stress [57]. In addition, malonylation of mTOR at K1218 attenuates its kinase activity in FASN-depleted endothelial cells and contributes to vascular defects [58]. Therefore, these diverse PTMs confer flexible regulation of mTOR signaling in response to environmental inputs.

### 2.2. mLST8

As the shared subunit of the mTORC1 and mTORC2 complexes, mLST8 was initially identified to bind to the mTOR kinase domain and stabilize mTOR interaction with Raptor, leading to enhanced mTORC1 activity toward S6K1 and 4E-BP1 [59]. However, loss of mLST8 only abrogates the integrity and activity of mTORC2, but not mTORC1 in mice [60,61]. A recent study revealed that TRAF2/OTUD7B-modulated K63-linked polyubiquitination of mLST8 at K305/K313 serves as a switch to govern the balance between mTORC2 and mTORC1. Specifically, mLST8 ubiquitination by TRAF2 disrupts mTORC2 complex integrity by precluding Sin1 and Rictor binding to mTOR, and on the other hand, it promotes mTORC1 complex formation through enhancing mTOR interaction with Raptor. As a result, loss of mLST8 ubiquitination by either mutating the K305/K313 or depleting the E3 ligase TRAF2 enhances mTORC2 activation and tumor growth, while knockout of the deubiquitinase OTUD7B exhibits opposite phenotypes [62]. These studies indicate that mLST8 is indispensable for mTORC2 activation and tumor growth. In addition, *mLST8* knockout mice are defective in vascular development [60], indicating it may be involved in vascular diseases. 

### 2.3. DEPTOR

DEPTOR, also known as DEPDC6, is an mTOR-interacting protein that serves as an endogenous inhibitor of both mTORC1 and mTORC2. DEPTOR depletion enhances mTOR signaling, cell growth, and survival. In contrast, DEPTOR overexpression blocks mTORC1 activation but unexpectedly activates mTORC2 signaling through relieving the mTORC1-mediated inhibitory feedback signal to the PI3K/mTORC2/Akt pathway [12]. Generally, DEPTOR expression is frequently decreased in most cancers but is upregulated in multiple myeloma, thyroid cancer, and lung cancer [63]. Thus, DEPTOR can be an oncogene or tumor suppressor. Interestingly, mTOR phosphorylates DEPTOR at 13 S/T residues in the linker between the DEP domain and PDZ domain. This phosphorylation event collaborates with casein kinase I to generate a degron that can be recognized by the E3 ubiquitin ligase SCF^β-TRCP^, resulting in its ubiquitination and degradation [64,65,66]. Moreover, p38γ or p38δ-mediated phosphorylation of DEPTOR also induces its degradation, leading to heart hypertrophy [67]. These studies suggest that aberrant DEPTOR expression contributes to the deregulation of mTOR signaling in human diseases.

## 3. Regulation of Components in mTORC1 Pathway by PTMs

### 3.1. Raptor

Raptor is the unique subunit of the mTORC1 complex and acts as a scaffolding protein to determine mTORC1 activity and substrate specificity [4,5,9,10]. Studies showed that conditional ablation of *Raptor* in mouse leads to various tissue-specific diseases, such as heart failure [68], hepatic steatosis [69], and muscle atrophy [70]. Unexpectedly, liver-specific *Raptor*-knockout promotes liver cancer development [71]. Similar to mTOR, extensive studies focused on the role of phosphorylation in regulating Raptor for mTORC1 activation. In response to upstream inputs such as insulin, amino acids, and energy, Raptor is phosphorylated at S863 and other multiple residues (S696, T706, S855, S859, and S877), deficiency of which leads to a reduction in mTOR kinase activity [72]. JNK, RSKs, and cdc2 have been reported to directly phosphorylate these sites under different conditions including osmotic stress, MAPK signaling activation, and mitosis [73,74,75,76,77]. Another important phosphorylation event on Raptor occurs under energy stress. Specifically, the cellular energy sensor AMPK directly phosphorylates Raptor at two well-conserved serine residues S722 and S792, which induces Raptor interaction with 14-3-3 protein, leading to mTORC1 inactivation and cell cycle arrest. Therefore, phosphorylation of S722/S792 serves as a checkpoint to coordinate cell growth with energy status [78]. Recently, Raptor was reported to be directly phosphorylated at S606 by the Hippo pathway core component LATS kinase in response to cellular inhibitory signals, resulting in attenuation of the mTORC1 kinase activity. This study provides a direct connection between the mTORC1 and the Hippo pathways to coordinately dictate cell growth, cell proliferation, and organ growth [79]. Other PTMs including ubiquitination and acetylation of Raptor also play critical roles on mTORC1 activation. For example, DDB1/CUL4-mediated polyubiquitination of Raptor enhances the stability and activity of the mTORC1 complex, which can be antagonized by the ubiquitin hydrolase UCH-L1 [80,81]. Sung et al. reported that acetyl-coenzyme A, the metabolite of leucine, can induce the EP300-depedent acetylation of Raptor. As a result, leucine deprivation decreases Raptor acetylation, leading to mTORC1 inhibition and autophagy induction [82].

### 3.2. PRAS40

In 2007, three independent groups identified PRAS40 as a Raptor-interacting protein that acts as an inhibitor to block insulin/Rheb-mediated activation of mTORC1 signaling [83,84,85]. In response to growth factors stimulation, PRAS40 is phosphorylated on multiple residues, which promotes its binding to 14-3-3 protein and dissociation from the mTORC1 complex. Several kinases have been reported to participate in PRAS40 phosphorylation including Akt and PIM1 for T246, mTORC1 for S183 and S221, and PKM2 for S202/203 [86]. Importantly, elevated PRAS40-T246 phosphorylation was observed in several cancer types including malignant melanoma [87], prostate cancer [88], gastric cancer [89], and NSCLC [90]. Moreover, induction of PRAS40-T246 phosphorylation enhances insulin sensitivity in obese patients with type 2 diabetes [91]. In addition, PRAS40 has protective roles in neurodegenerative diseases and cardiovascular diseases [92]. Therefore, phosphorylation of PRAS40-T246 can be used as a biomarker for predicting the efficacy of inhibitors targeting the Akt/mTOR pathway in human diseases.

### 3.3. TSC Complex

TSC complex, composed of TSC1, TSC2, and TBC1D7, is a negative regulator of mTORC1 signaling [93]. Mutations or loss of TSC1/2 lead to hyperactivation of mTORC1 signaling and have been associated with many human disorders, such as lymphangioleiomyomatosis (LAM) and autism [94]. The TSC complex possesses GAP activity to convert Rheb from the active GTP-bound form to the inactive GDP-bound form [95]. Studies showed that in response to growth factor stimulation, phosphorylation of TSC at several sites by multiple kinases inhibits its GAP function and subsequently, relieves its inhibition on mTORC1 signaling, including Akt-mediated phosphorylation of S939/T1462, ERK-mediated phosphorylation of S664, and RSK1-mediated phosphorylation of S1798 [25,26,96,97,98]. In contrast, energy deprivation induces AMPK-dependent phosphorylation of TSC2 at S1387 and enhances TSC GAP activity, which in turn inactivates mTORC1 and protects cells from apoptosis [99]. These phosphorylation events represent key mechanisms for growth factors- or energy-regulated mTORC1 activation. In addition, IKKβ-mediated phosphorylation of TSC1 at S487/S511 leads to TSC1 inhibition and mTORC1 activation, which promotes angiogenesis and tumor development [100]. Notably, TSC1/2 are ubiquitinated and degraded by multiple E3 ligases including TRIM31 [101], Pam [102], E6AP [103], and DDB1-CUL4-ROC1 [104], resulting in mTORC1 activation.

### 3.4. Rheb GTPase

Rheb is a small GTPase that is enriched on multiple endomembrane compartments including the lysosomal membrane where it interacts with and activates mTORC1. Farnesylation of Rheb in the C-terminal CaaX motif is required for its lysosomal localization [105,106]. In contrast, PRAK-mediated S130 phosphorylation inhibits GTP loading on Rheb under energy-deficient conditions, providing an AMPK-independent mechanism for mTORC1 inhibition by energy stress [107]. The nucleotide-loading status of Rheb is also regulated by RNF152-catalyzed ubiquitination at K8, which keeps Rheb in the inactive GDP-bound form to suppress mTORC1 activation [108]. A recent study showed that amino acids promote Rheb polyubiquitination to enhance its interaction with mTORC1. Interestingly, the ubiquitinated Rheb is subsequently degraded due to the loss of protection by the ATXN3 deubiquitinase that is dissociated from the lysosome by the active Rag GTPases [109]. Rheb functions as an oncogene, which is overexpressed in many cancers including prostate cancer and liver cancer [110]. Moreover, dysregulation of Rheb has also been linked to neurodegenerative diseases such as Parkinson’s disease (PD) and AD [111]. Therefore, Rheb and its regulatory PTM enzymes are potential therapeutic targets.

### 3.5. Rag GTPases

The activation of the Rag heterodimer is the key step for mTORC1 lysosomal recruitment and activation. Notably, RagC is frequently mutated in follicular lymphoma [112] and the RagC-S75Y mutation is associated with the development of syndromic fetal dilated cardiomyopathy [113]. Several lines of evidence demonstrate that PTMs play a critical role in timely controlling Rags activation by amino acids. K63-linked polyubiquitination of RagA by E3 ligases RNF152 and Skp2 serves as an anchor to facilitate recruitment of GATOR1 and RagA^GTP^ hydrolysis, leading to inactivation of mTORC1 signaling [114,115]. However, the specific mechanisms are different in these studies. Deng et al. showed that amino acid starvation induces RNF152-mediated polyubiquitination of RagA at four sites (K142/K220/K230/K244) and *RNF152* knockout leads to hyperactivation of mTORC1 [114]. In contrast, Jin et al. demonstrated that amino acid stimulation promotes Skp2-dependent RagA polyubiquitination, which acts as a negative feedback signal to terminate amino acid-induced mTORC1 activation [115]. However, whether and how RNF152 and Skp2 coordinately regulate RagA polyubiquitination in response to amino acid remain elusive. Interestingly, insulin induces mTORC1-dependent phosphorylation of RagC at three highly conversed sites (S2, S21, and T394). Deficiency in this phosphorylation attenuates mTORC1 maximal activation by altering mTOR and Raptor interaction. This study reveals another crosstalk between amino acid and growth factor-stimulated mTORC1 signaling [116].

### 3.6. Ragulator Complex

Ragulator interacts with Rag GTPases and is necessary for localizing the Rag proteins and mTORC1 to the lysosomal surface for activation. Among the five components of the Ragulator complex, myristoylation and palmitoylation in the N-terminus of p18 serve as a membrane localization signal for tethering Ragulator to the lysosomal surface [117]. Moreover, the protein stability of p18 is controlled by the E3 ubiquitin ligase UBE3A through the ubiquitination-proteasome system [118]. As imbalance in UBE3A is associated with Angelman syndrome and autism [119,120], this finding indicates that aberrant expression of p18/Ragulator may contribute to neurological disorder. A structural study also identified that C7orf59 is phosphorylated at S67 by PKA, which disrupts its interaction with p18. However, it is unknown whether S67 phosphorylation is regulated by amino acid and controls mTORC1 signaling [121].

### 3.7. GATOR1, GATOR2, and KICSTOR Complexes

The GATOR1 complex is composed of three subunits including DEPDC5, NPRL2, and NPRL3, while the GATOR2 complex consists of WDR24, WDR59, MIOS, SEH1L, and SEC13 [29]. KISCTOR was recently identified as a four-protein complex containing SZT2, KPTN, ITFG2, and C12orf66 [30,31]. These three complexes form a huge complex to regulate amino acid-mediated mTORC1 signaling. Specifically, SZT2 serves as the platform to directly connect the other three components of KISCTOR and GATOR1 to the lysosomal membrane, while GATOR2 directly interacts with and inhibits GATOR1. Of note, the integrity of these complexes and their interactions are not affected by amino acids. Therefore, GATOR1 and KICSTOR are two negative regulators, whereas GATOR2 is a positive regulator of mTORC1 lysosomal localization and activation. To date, only DEPDC5 has been reported to be regulated by PTMs. Chen et al. showed that DEPDC5 is K48-linked and polyubiquitinated at multiple lysine residues by the E3 ubiquitin ligase KLHL22 upon amino acid stimulation and consequently decreases the DEPDC5 protein levels and activates mTORC1 signaling. Consistently, elevated KLHL22 expression is positively correlated with decreased DEPDC5 protein levels in tumor samples from breast cancer patients [122]. Pim and Akt kinases-mediated phosphorylation of DEPDC5-S1530 results in activation of mTORC1 in an amino acid-insensitive fashion. As a result, S1530A mutant significantly suppresses cell growth and tumorigenesis in part due to the deficiency in mTORC1 signaling [123]. However, the molecular mechanism underlying how DEPDC5 phosphorylation promotes mTORC1 activation is unknown. Recently, mutations in the GATOR1 subunits and SZT2 are linked to epilepsy [124,125]. Thus, it will significantly expand our knowledge on mTORC1 pathway regulation if we can reveal more PTMs on these complexes. 

### 3.8. FLCN

FLCN is considered as a tumor suppressor, mutations in which are the drivers of Birt–Hogg–Dubé hereditary cancer syndrome [126]. Moreover, cardiac-specific knockout of *FLCN* leads to cardiac hypertrophy and dysfunction in mice [127]. FLCN functions as a GAP for RagC/D^GTP^ hydrolysis in the presence of amino acids [21]. FLCN was reported to be phosphorylated at multiple residues including S62/S73 by mTORC1 [128], S406/S537/S542 by ULK1 [129], and S302 by unknown kinase [130]. FLCN has also been found to be ubiquitinated at K206/K559 by mass spectrometric analysis [131,132]. However, whether phosphorylation and ubiquitination of FLCN regulate mTOR signaling have not yet been investigated. 

### 3.9. Amino Acid Sensors

To date, three types of cytosolic amino acid sensors for the mTORC1 pathway have been identified: the leucine sensor Sestrin2, arginine sensor CASTOR1, and S-adenosylmethionine (SAM) sensor SAMTOR [133]. Mechanically, in the absence of leucine, Sestrin2 interacts with GATOR2 to release its inhibition on GATOR1, and consequently inactivates Rags-mediated mTORC1 signaling. Conversely, in the presence of leucine, Sestrin2 binds to leucine and dissociates from GATOR2, leading to GATOR2-dependent activation of mTORC1 signaling [33]. CASTOR1 applies the same mechanism as Sestrin2 to regulate arginine-induced mTORC1 activation [35], while SAMTOR directly interacts with GATOR1 to regulate SAM-mediated mTORC1 regulation [34]. Emerging studies demonstrate that phosphorylation and ubiquitination of Sestrin2 play a critical role in regulating mTORC1 signaling. ULK1-mediated phosphorylation of Sestrin2 at S73/S254 enhances its interaction with GATOR2, leading to mTORC1 inhibition and autophagy induction [134,135]. The RING-type E3 ligase RNF186 promotes ubiquitination and degradation of Serstrin2, leading to hyperactivation of the mTORC1 pathway [136]. Notably, RNF186-A64T mutation is a risk factor for the development of ulcerative colitis (UC), a kind of inflammatory bowel disease (IBD) [137]. It will be interesting to investigate whether Sestrins2/mTORC1 signaling is involved in RNF186-A64T-associated UC. Moreover, the expression of Sestrins2 is induced by stress, such as oxidative stress, genotoxic stress, and hypoxia, and exerts a protective role in cardiovascular and neurodegenerative diseases [138]. 

### 3.10. mTORC1 Downstream Targets 

As a S/T protein kinase, mTORC1 exerts its biological functions on cell growth and metabolism by phosphorylating multiple downstream substrates, most of which have been demonstrated to play a role in various types of human diseases, such as cancers and cardiovascular and neurodegenerative diseases [3]. Here, we review the roles of PTMs on three critical mTORC1 downstream substrates.

The S6K kinases (S6K1 and S6K2) are critical downstream effectors of mTORC1 in controlling fundamental cellular processes, such as transcription, translation, metabolism, and cell growth [139]. Phosphorylation is the key mechanism regulating S6K1 activation [140]. In response to growth factors and nutrients, S6K1 is specifically phosphorylated at T389 by mTORC1 [141], followed phosphorylation at T229 by PDK1 [142,143]. Sequential phosphorylation at these two sites is essential for S6K1 full activation. In addition, phosphorylation at multiple sites in the C-terminal region also contributes to S6K1 activity [144]. Aside from phosphorylation, acetylation of S6K1 at the C-terminus by p300/PCAF attenuates mTORC1-dependent S6K1 phosphorylation [145], and polyubiquitination of S6K1 by E3 ubiquitin ligase ROC1 promotes its degradation [146]. 

ULK1, a gate keeper for autophagy initiation, is a key downstream effector mediating the role of mTORC1 signaling in autophagy. Upon glucose starvation, AMPK activates ULK1 by directly phosphorylating S317/S377 to induce autophagy. Conversely, under nutrient- and energy-rich conditions, mTORC1 directly phosphorylates ULK1 at S757 to disrupt ULK1 interaction with AMPK, leading to autophagy inhibition [147]. Recently, ULK1 was reported to be O-GlcNAcylated on T754 by OGT under glucose starvation, which cooperates with mTORC1/AMPK to induce autophagy [148]. Acetylation of ULK1 at K162/K606 by acetyltransferase TIP60 is also required for ULK1-mediated autophagy in response to growth factors withdrawal [149]. In addition, mTORC1/AMBRA1 coordinately regulates E3 ubiquitin ligase TRAF6-mediated K63-linked polyubiquitination of ULK1, which enhances ULK1 stability and function [150]. These studies suggested that mTORC1-mediated phosphorylation of ULK1 plays a key role in coordinating with other PTMs to control ULK1 activation and autophagy induction.

Grb10 is an adapter protein that interacts with tyrosine kinase receptors, such as insulin receptor [151]. mTORC1-mediated phosphorylation of Grb10 at S501/S503 increases its stability. Since Grb10 is a negative regulator of insulin signaling, Grb10 accumulation activates the negative feedback loop from mTORC1 to the PI3K/Akt pathway, leading to inactivation of PI3K/Akt signaling [128]. 

## 4. Regulation of Components in mTORC2 Pathway by PTMs

### 4.1. Rictor 

In searching the mTOR-interacting proteins, Rictor was identified as a novel mTOR-associated component, which also binds to mLST8 and confers a rapamycin-insensitive feature to mTORC2 [7]. Acetylation and phosphorylation are two major modifications of Rictor. p300 promotes acetylation of Rictor at multiple sites including K1092, K1095, K1116, K1119, and K1125, which enhances mTORC2 kinase activity toward Akt in response to growth factor stimulation [152]. Notably, phosphorylation of Rictor by GSK3 at T1695 suppresses mTORC2 signaling through different mechanisms. T1695 phosphorylation primes for FBXW7-mediated ubiquitination and degradation of Rictor [153], which may account for the overexpression of Rictor in cancers [154]. S1235 phosphorylation impairs mTORC2 binding to Akt in vitro [155]. Rictor was also found to be phosphorylated at T1135 by S6K1. Mutating T1135 to alanine enhances Akt signaling induced by growth factors, although neither mTORC2 kinase activity and integrity nor cellular localization were affected by T1135 phosphorylation [156]. Interestingly, the total levels of Rictor and T1135 phosphorylation are downregulated in AD [157]. 

### 4.2. Sin1 

Later studies characterized Sin1 as another essential component of the mTORC2 complex, the depletion of which abolishes mTORC2 complex formation [11,158]. Sin1 is frequently overexpressed in many cancer types and plays a critical role in cell proliferation, epithelial mesenchymal transition (EMT), and metastasis [159,160]. Sin1 phosphorylation has been found to have different impacts on mTORC2 activation. Dual phosphorylation of Sin1 at T86/T398 by S6K1 or Akt dissociates Sin1 from mTORC2, leading to the inhibition of mTORC2 [161], whereas phosphorylation of Sin1 at T86 alone enhances mTORC2 kinase activity [162]. Therefore, Sin1 phosphorylation provides a delicate regulation of mTORC2 activation. Recently, Cui et al. reported that the CUL5–SOCS6 complex promotes Sin1 ubiquitination at K162, K276, and K302, leading to Sin1 degradation and mTORC2 inactivation [163].

### 4.3. PI3K and PTEN

It has been reported that the Sin1-PH domain binds to PIP3 and subsequently releases its inhibition on the mTOR kinase domain, leading to mTORC2 activation in response to growth factors [40]. Thus, the production of PIP3 is a critical step for mTORC2 activation. The PI3Ks are the lipid kinases that phosphorylate PIP2 at the plasma membrane to generate PIP3, which is antagonized by the lipid phosphatase PTEN. PI3Ks are frequently mutated or amplified, whereas PTEN is commonly deleted or mutated in many human diseases including but not limited to cancers, metabolic disorders, and AD [164]. In addition to genetic alterations, a number of studies have also demonstrated that PTMs including SUMOylation, phosphorylation, ubiquitination, and acetylation play important roles in regulating the activity and stability of PI3K and PTEN [165,166,167,168], which may play a role in regulating mTORC2 activation. 

### 4.4. mTORC2-Specific GTPases 

Like mTORC1, several small GTPases have been identified recently to directly regulate mTORC2 kinase activity. The Rho family of GTPase Rac1 directly binds to mTOR through the C-terminal region and promotes the activity of both mTORC1 and mTORC2 independent of its nucleotide binding states and PI3K pathway [41]. Of the Ras family of GTPases, Ras and Rit promote mTORC2 activation through interaction with Sin1 [169,170]. Despite multiple types of PTMs being reported to control the activation and cellular localization of Rac1 and Ras [171,172], it is unknown whether these PTMs are involved in regulating mTORC2 kinase activity. Fortunately, studies in Dictyostelium have provided more detailed knowledge on GTPase-mediated activation of mTORC2. The small GTPases, Rap1 and RasC, bind to Sin1 and the mTOR kinase domain, respectively, which cooperatively activate mTORC2 in response to chemoattractants [173]. Interestingly, GDP-bound RacE interacts with both Tor (mTOR homologue) and PiaA (Rictor homologue). Moreover, phosphorylation of RacE^GDP^ at S192 by GSK3 mediates its interaction with RasC^GTP^ to promote mTORC2 activation [42]. These studies suggest that GTPases may control mTORC2 activity in a fashion similar to mTORC1.

### 4.5. mTORC2 Downstream Effectors

As a major substrate, Akt is phosphorylated at S473 by mTORC2 [174], which cooperates with PDK1-mediated T308 phosphorylation to promote PI3K-dependent activation of Akt in response to growth factors [39]. Furthermore, many other PTMs have been identified to fine tune Akt kinase activity (review in [175]). Phosphorylation at S477/S479 by the cyclin A-CDK2 complex along with mTORC2-mediated S473 phosphorylation enhances Akt activation in a cell cycle-dependent manner [176]. K63-linked polyubiquitination of Akt in the PH domain by E3 ubiquitin ligases TRAF6 [177] and Skp2 [178] promotes Akt membrane localization and activation, leading to enhanced glycolysis and tumorigenesis. Recently, two groups independently demonstrated that SETDB1-mediated methylation of Akt at K64 or K140/K42 crosstalks with PI3K and TRAF6/Skp2 to promote Akt activation and tumor growth [179,180]. Interestingly, hydroxylation of Akt at P125/P313 by the prolyl hydroxylase EglN1 triggers its interaction with E3 ubiquitin ligase VHL, leading to Akt inhibition independent of its ubiquitination [181]. As these PTM enzymes are frequently deregulated, these studies provide multiple mechanisms for hyperactivation of Akt in cancers, metabolic disorders, and other diseases [182]. 

Besides Akt, PKCs and SGK1 are also the downstream effectors of mTORC2. Phosphorylation of PKCα by mTORC2 in the turn motif promotes its maturation, stabilization, and activation, which controls actin polymerization, cell shape, and mobility [60,183,184]. Moreover, other PTMs including ubiquitination, O-GlcNAc, tyrosine nitration, and tyrosine phosphorylation also play a role in regulating PKC functions [185,186]. Phosphorylation of SGK1 at the hydrophobic motif by mTORC2 promotes its activation [187]. SGK1 is polyubiquitinated and rapidly turned over by different E3 ubiquitin ligases including CHIP, HRD1, NEDD4L, and Rictor/Cullin 1 [188].

## 5. Targeting mTOR Signaling for Treating Human Diseases

### 5.1. mTOR Specific Inhibitors

Dysregulation of mTOR signaling is tightly associated with many human diseases, such as obesity, diabetes, cancers, and neuronal disorders [3], making mTOR an ideal therapeutic target. Extensive efforts have focused on the development of mTOR inhibitors. Rapamycin and its derivatives (termed as Rapalogs) are the first generation of mTOR inhibitors. Rapamycin was originally identified as an antifungal and immunosuppressive drug and was approved by the FDA to use for preventing allograft rejection in transplantation and restenosis in coronary artery stents, but not for cancer treatment due to its poor solubility [189]. To date, several water-soluble Rapalogs have been developed and are evaluated in many clinical trials. Notably, Temsirolimus and Everolimus have been approved by the FDA for treating advanced renal cell carcinoma [190]. However, their anti-tumor efficacy is modest and Rapalog resistance is frequently acquired in the majority of solid tumors. One of the causes is that Rapalogs only partially inhibit mTORC1 but not mTORC2 [191]. To resolve this drawback, the secondary generation of ATP-competitive mTOR inhibitors (TORKi) has been developed to target both mTORC1 and mTORC2. Preclinical studies showed that these pan-mTOR inhibitors can overcome Rapalog resistance and are currently being evaluated in clinical trials [192,193,194]. However, an emerging study demonstrated that acquired mutations in mTOR result in hyperactivation of mTOR kinase and confer resistance to both rapalogs and TORKi [195]. To overcome the resistance, a third generation of mTOR inhibitor was generated by crosslinking rapamycin with a TORKi named Rapalink [195]. Importantly, Rapalink exhibits better anti-tumor efficacy than rapamycin or TORKi in glioblastoma [196]. 

### 5.2. Dual PI3K/mTOR Inhibitors

Despite TORKi being able to block mTORC2-dependent phosphorylation of Akt-S473, its inhibition on mTORC1 may release the negative feedback regulation of PI3K/PDK1/Akt signaling [197,198], consequently attenuating the efficacy or acquiring resistance to TORKi [199]. Given the similarity of structural kinase domain between PI3K and mTOR, compounds targeting both kinases have been developed. Notably, Dactolisib (BEZ235 and NVP-BEZ235) was the first dual PI3K/mTOR inhibitor entering clinical trials. However, limited anti-tumor effect and poor tolerance have been reported in the treatment of prostate cancer, pancreatic neuroendocrine tumor, advanced breast cancer, and advanced renal cancer [200,201,202]. Currently, several clinical trials of dual PI3K/mTOR inhibitors including GDC-0980 (Apitolisib) and PF-04691502 and PF-05212384 (Gedatolisib) either in monotherapy or in combination therapies are ongoing [203]. Since PI3K and the mTOR pathway are critical for cell growth and metabolism, inhibition of both may increase toxicity and adverse effects. Therefore, balance between the anti-tumor activity and dose-tolerability has to be seriously considered in clinical trials with these dual PI3K/mTOR inhibitors. 

### 5.3. Akt Inhibitors

As the major downstream effector of PI3K/mTOR signaling, Akt plays a central role in promoting cell survival and apoptosis. Given hyperactivation of Akt is a hallmark of cancers, targeting Akt for cancer therapy has been extensively investigated [39]. Many Akt inhibitors including AZD5363, MK2206, and GDC-0068 are in phase I/II clinical trials, especially for breast cancers [204,205,206]. However, none of these inhibitors have achieved satisfactory outcomes in monotherapy and entered phase III clinical trials, which is largely due to the complexity of Akt signaling and toxicity. In the human genome, Akt has three isoforms that possess different localizations, substrates, and biological functions [207]. Thus, development of Akt isoform-specific inhibitors may be helpful to tackle complicated Akt signaling. In addition, the combination of Akt inhibitors with chemotherapy and other targeted therapy is an option for reducing toxicity [208].

### 5.4. Targeting mTOR Signaling Regulators 

As the key events in signaling transduction, PTMs including phosphorylation, ubiquitination, methylation, and acetylation, among others, provide a tight and reversible regulation of mTOR signaling. Aberrancies in these PTM-related enzymes may lead to the aberrant activation of the mTOR pathway. Therefore, targeting these mTOR-related regulators would offer an option for treating mTOR-associated human diseases. Below, we primarily focus on kinases, E3 ubiquitin ligases, and deubiquitinases, which are key regulators of the mTOR pathway.

#### 5.4.1. Kinases

As S/T kinases, RSKs regulate diverse cellular processes by phosphorylating many downstream substrates [209]. Notably, RSKs promote cell growth and protein synthesis by activating the mTORC1 pathway through phosphorylating both TSC2 and Raptor as described previously. Of note, hyperactivation of RSKs has been implicated in various types of diseases, such as cancers, cardiovascular diseases, and liver and kidney diseases. Several pan-RSK inhibitors have been developed and have shown promising results in preclinical models [210]. SL0101 significantly suppresses breast cancer cell proliferation and tumor growth. BIX02565 reduces cardiac ischemia/reperfusion injury, and BI-D1870 attenuates autoimmune encephalomyelitis in mice. However, further studies and more specific and efficient RSK inhibitors are needed to target RSKs for clinically treating human diseases. 

JNKs including JNK1, JNK2, and JNK3 in mammals are predominantly activated by environmental stress and regulate many physiological processes, such as cell proliferation, apoptosis, immune response, inflammation, and neuronal plasticity [211]. Their hyperactivation is associated with a multitude of diseases including cancers, diabetes, autoimmune disease, and neurological disorders, particularly AD [212]. Thus, JNKs are potential therapeutic targets. A number of JNK inhibitors have been extensively investigated in preclinical models of human diseases and several of them have entered clinical trials [213]. However, most of the trials seem to achieve limited success largely due to sequence similarity but also the functional diversity of the three JNKs in different tissues/diseases. Therefore, isoform-specific JNK inhibitors would facilitate their clinical applications. 

The PIM kinase family containing three members, PIM1–3, and plays a critical role in cell proliferation, apoptosis, and migration by directly phosphorylating a number of downstream targets, such as MYC, CDC25, p21/p27, and TSC2. Overexpression of PIM kinases has been observed in a variety of human cancers and is associated with poor prognosis and drug resistance in prostate cancer and breast cancer [214]. As there have been significant advances in understanding the oncogenic role of PIM kinase in the past decade, several promising PIM kinase inhibitors have been developed and evaluated in clinical trials for prostate cancer, acute myelogenous leukemia, and multiple myeloma [215]. Notably, pan-PIM inhibitor LGH447 was generally well tolerated and exhibited a promising therapeutic outcome in multiple myeloma patients. Moreover, clinical trials for a combination of LGH447 with PI3K inhibitor (BYL719) or CDK4/6 inhibitor (LEE011) are ongoing [216]. 

Other mTOR pathway-associated kinases including cdc2 [217], IKKβ [218], AMPK [219], LATS [220], ULK1 [221], and GSK3 [222] have been implicated in human diseases. AMPK plays a central role in maintaining cellular energy homeostasis and its inactivation may lead to metabolic disorders including diabetes, obesity, liver, and kidney diseases. Thus, pharmacological activation of AMPK has gained extensive attention in the past five years. A number of direct or indirect AMPK activators have been approved for use or are in clinical trials [219]. ULK1 is a critical S/T protein kinase for autophagy initiation, which is a substrate and a negative regulator of mTORC1. ULK1-mediated autophagy can either promote tumor progression or suppress tumor growth, as well as confer drug resistance [221]. ULK1 inhibitors including ULK-101, MRT68921, and SBI-0206965 significantly suppress cancer cell proliferation and induce apoptosis [223,224], which is worth further investigation in mice models. 

#### 5.4.2. E3 Ubiquitin Ligases and Deubiquitinases (DUBs)

As described above, a dozen E3 ligases and DUBs have been identified to regulate mTOR signaling by controlling protein stability, enzymatic activity, and complex formation. Aberrancies in these E3s/DUBs are frequently observed in human diseases, which may be in part responsible for the dysregulation of mTOR signaling. 

FBXW7, a cullin-based SCF type of E3 ubiquitin ligase, plays an important role in various biological processes such as cell cycle transition, cell proliferation, apoptosis, metastasis, and immune response [225]. FBXW7 is a well-acknowledged tumor suppressor that promotes the degradation of multiple oncogenic proteins such as cyclin E, MCL1, c-Myc, mTOR, and Rictor. Consistently, FBXW7 is frequently lost or mutated in various types of cancers. Notably, more than 30% of T cell acute lymphoblastic leukemia and cholangiocarcinomas harbor *FBXW7* mutations [225]. Beside cancers, loss of FBXW7 also contributes to other mTOR-related diseases, such as metabolic disorders and aging, which warrants further studies. 

Parkin belongs to the RING-between-RING (RBR) family of E3 ubiquitin ligases that regulate a variety of cellular processes including mitochondrial quality control, anti-oxidative stress, and mitophagy. Mutations in Parkin lost its protective role on the survival of dopaminergic neurons, which is the major cause of autosomal recessive parkinsonism. Notably, mTOR signaling exerts either a neuroprotective or neurotoxic role depending on PD models and has been linked to PD. It will be interesting to investigate whether Parkin-mediated ubiquitination and activation of mTOR signaling play a role in PD. Moreover, Parkin is an emerging tumor suppressor as it is frequently lost in cancers including breast, lung, colorectal, and ovarian cancers [226]. Consistently, Parkin knockout mice develop hepatocellular carcinoma [227]. However, the exact mechanisms underlying how Parkin suppresses tumor growth is largely unknown. It is possible that Parkin-mediated regulation of mTOR signaling contributes to its tumor suppressive function.

Other E3 ubiquitin ligases including Skp2 [228] and KLHL22 [122] are oncoproteins, which are overexpressed in many cancers. Several Skp2 inhibitors including compound 25 [229], compound A [230], and compound C [231] exhibit significant anti-growth/tumor effects in cancer cells and mouse models. In contrast, the E3 ligase RNF152 suppresses both amino acids and growth factors-induced mTORC1 activation by inhibiting the critical GTPases RagA and Rheb, respectively. Downregulation of RNF152 results in the hyperactivation of mTORC1, which has been observed in a variety of cancers including colon, lung, kidney, and liver cancers [232]. TRAF2 is a crucial inflammatory regulator that activates multiple signaling pathways including NF-κB, MAPKs, and IRFs. Upregulation of TRAF2 has been linked to multiple inflammatory diseases, such as atherosclerosis, AD, PD, and multiple sclerosis [233]. In addition to inflammation, TRAF2 also plays a tumor suppressive or oncogenic role in different types of cancers [234]. 

OTUD7B belongs to the ovarian tumor (OUT) family of deubiquitinases and prefers to hydrolyze the K63-linked ubiquitin chain [235]. OTUD7B has been demonstrated to have a crucial role in inflammatory and immune response through the NF-κB pathway [236,237]. Recent studies suggested that OTUD7B may promote cancer progression by activating oncogenic pathways including EGFR and mTORC2/Akt signaling [62,238]. Consistently, upregulation of OTUD7B positively correlates with cancer progression and poor prognosis in lung squamous carcinoma and adenocarcinoma [239]. Another DUB, UCH-L1, is highly expressed in neurons and has been implicated in PD and AD [240]. 

Despite the dramatic advance in understanding the roles and regulations of E3 ubiquitin ligases/DUBs in human diseases, only a few of their inhibitors are clinically used or in clinical trials [241]. In the past five years, a revolutionary technology called proteolysis targeting chimeras (PROTACs) has gained great attention. Mechanically, a PROTAC is a small molecule composed of two parts for binding of a E3 ligase and a specific protein, leading to protein degradation [242]. To date, several E3 ligases have been used for designing PROTAC, including Hrt1, VHL, CRBN, MDM2, DCAF15, RNF114, and IAPs [243]. Recently, two PROTAC molecules, ARV-110 and ARV-471, have entered phase I clinical trials for prostate cancer and breast cancer, respectively [244]. It is expected that the PROTAC technology will significantly facilitate targeting of the disease-driving proteins, especially the “undruggable” proteins for human diseases treatment in the next decade. 

## 6. Conclusions

Since identification of the mTOR kinase in the 1990s, significant progress has been made in the mTOR research field, notably mTORC1 signaling. Many new components and regulators were discovered to make up the delicate mTOR network. Given the essential role of the mTOR pathway in many fundamental biological processes, complete loss of most mTOR components has been proved to be embryonically lethal in mouse, such as mTOR, Raptor, mLST8, Rictor, Sin1, Rheb, and RagA [245]. Therefore, aberrant mTOR signaling in human diseases may be caused by tissue-specific deficiency in these components in terms of their expression, activity, localization, or protein interaction, all of which can be regulated by PTMs. As described in this review, PTMs indeed offer a flexible and precise regulation of the mTOR signaling in response to various external inputs. Moreover, aberrant expression and enzymatic activity of these PTM enzymes are frequently tissue-dependent, which may explain the imbalance of mTOR activity in different diseases. 

To date, more than 200 types of PTMs have been identified and play key roles in regulating protein functions in all signaling pathways. However, only a few kinds of PTMs have been validated in a dozen mTOR components and linked to the dysregulation of mTOR signaling. Therefore, identifying functional PTMs on mTOR components would be of great interest in future studies, which may provide more strategies for therapeutically targeting mTOR signaling in clinic.

## Figures and Tables

**Table 1:** Major post-translational modifications (PTMs) of the mTOR signaling pathway.

Target	PTM	Sites	Enzyme	Influence on mTOR	References
mTOR	phosphorylation	S2448	AktS6K1	activation	[43,44,45,46]
mTOR	phosphorylation	S2446	AMPK	inhibition	[47]
mTOR	phosphorylation	S1261	PI3K/TSC	activation	[48]
mTOR	phosphorylation	S2481	mTOR	activation	[49,50,51]
mTOR	phosphorylation	S2159/T2164	unknown	activation	[52]
mTOR	ubiquitination	unknown	FBXW7	inhibition	[55,56]
mTOR	ubiquitination	K2066/K2306	Parkin	activation	[57]
mTOR	malonylation	K1218	unknown	inhibition	[58]
mLST8	ubiquitination	K305/K313	TRAF2	inhibition	[62]
mLST8	deubiquitination	K305/K313	OTUD7B	activation	[62]
DEPTOR	ubiquitination	unknown	SCF^β-TRCP^	activation	[64,65,66]
DEPTOR	phosphorylation	multiple S/T	p38γ or p38δ	activation	[67]
Raptor	phosphorylation	S696/T706/S863	JNK	activation	[73,74]
Raptor	phosphorylation	multiple S	cdc2	activation	[75,76]
Raptor	phosphorylation	S719/S721/S722	RSKs	activation	[77]
Raptor	phosphorylation	S722/S792	AMPK	inhibition	[78]
Raptor	phosphorylation	S606	LATS	inhibition	[79]
Raptor	ubiquitination	unknown	DDB1/CUL4	activation	[80]
Raptor	ubiquitination	unknown	UCH-L1	inhibition	[81]
Raptor	acetylation	unknown	EP300	activation	[82]
PRAS40	phosphorylation	T246	Akt or PIM1	activation	[86]
PRAS40	phosphorylation	S183/S221	mTORC1	activation	[86]
PRAS40	phosphorylation	S202/S203	PKM2	activation	[86]
TSC	phosphorylation	S939/T1462	Akt	inhibition	[25,26]
TSC	phosphorylation	S664	ERK	inhibition	[96,97]
TSC	phosphorylation	S1798	RSK1	activation	[98]
TSC2	phosphorylation	S1387	AMPK	inhibition	[99]
TSC1	phosphorylation	S487/S511	IKK β	activation	[100]
TSC1/2	ubiquitination	unknown	TRIM31	activation	[101]
TSC	ubiquitination	unknown	Pam	activation	[102]
TSC2	ubiquitination	unknown	E6AP	activation	[103]
TSC2	ubiquitination	unknown	DDB1/ROC1	activation	[104]
Rheb	farnesylation	unknown	unknown	activation	[105,106]
Rheb	phosphorylation	S130	PARK	inhibition	[107]
Rheb	ubiquitination	K8	RNF152	inhibition	[108]
Rheb	deubiquitination	unknown	ATXN3	inhibition	[109]
Rag A	ubiquitination	multiple lysine	RNF152	inhibition	[114]
Rag A	ubiquitination	unknown	Skp2	inhibition	[115]
Rag C	phosphorylation	S2/S21/T394	mTORC1	activation	[116]
p18	myristoylation	unknown	unknown	activation	[117]
p18	palmitoylation	unknown	unknown	activation	[117]
p18	ubiquitination	unknown	UBE3A	inhibition	[118]
C7orf59	phosphorylation	S67	PKA	unknown	[121]
DEPDC5	ubiquitination	multiple lysine	KLHL22	activation	[122]
DEPDC5	phosphorylation	S1530	Pim/Akt	activation	[123]
FLCN	phosphorylation	S62/S73	mTORC1	unknown	[128]
FLCN	phosphorylation	S406/S537/S542	ULK1	unknown	[129]
FLCN	phosphorylation	S302	unknown	unknown	[130]
FLCN	ubiquitination	K206/K559	unknown	unknown	[131,132]
Sestrin2	phosphorylation	S73/S254	ULK1	inhibition	[134,135]
Sestrin2	ubiquitination	K13	RNF186	inhibition	[136]
S6K1	phosphorylation	T389	mTORC1	activation	[141]
S6K1	phosphorylation	T229	PDK1	activation	[142,143]
S6K1	acetylation	C-terminal	p300/PCAF	inhibition	[145]
S6K1	ubiquitination	unknown	ROC1	inhibition	[146]
ULK1	phosphorylation	S317/S377	AMPK	activation	[147]
ULK1	phosphorylation	S757	mTORC1	inhibition	[147]
ULK1	O-GlcNAcylation	T754	OGT	activation	[148]
ULK1	acetylation	K162/K606	TIP60	activation	[149]
ULK1	ubiquitination	unknown	TRAF6	inhibition	[150]
Grb10	phosphorylation	S501/S503	mTORC1	inhibition	[128]
Rictor	acetylation	multiple lysine	p300	activation	[152]
Rictor	phosphorylation	T1695	GSK3	inhibition	[153]
Rictor	phosphorylation	S1235	Akt	inhibition	[155]
Rictor	phosphorylation	T1135	S6K1	activation	[156]
Sin1	phosphorylation	T86/T398	S6K1 or Akt	inhibition	[161]
Sin1	phosphorylation	T86	Akt	activation	[162]
Sin1	ubiquitination	multiple lysine	CUL5-SOC6	inhibition	[163]
Akt	phosphorylation	S477/S479	cyclin A/CDK2	activation	[176]
Akt	ubiquitination	unknown	TRAF6, Skp2	activation	[177,178]
Akt	methylation	K64/K140/K42	SETDB1	activation	[179,180]
Akt	hydroxylation	P125/P313	EglN1	inhibition	[181]
PKCα	phosphorylation	unknown	mTORC2	activation	[60,183,184]
SGK1	phosphorylation	unknown	mTORC2	activation	[187]

## Data Availability

No new data were created or analyzed in this study. Data sharing is not applicable to this article.

## Abbreviations

4E-BPs	eukaryotic initiation factor 4E-binding proteins
AMBRA1	autophagy and Beclin 1 regulator 1
AMPK	AMP-activated protein kinase
ATF4	activating transcription factor 4
ATG13	Autophagy-related protein 13
ATXN3	Ataxin-3
CAD	carbamoyl-phosphate synthetase 2
CASTOR1	cellular arginine sensor for mTORC1
CDC25	cell division cycle 25
CHIP	carboxy-terminus of Hsc70 interacting protein
CRBN	cereblon
CUL4	cullin 4
DCAF15	DDB1- and CUL4-associated factor 15
DDB1	DNA damage-binding protein 1
DEPDC5	DEP domain-containing 5
DEPTOR	DEP domain-containing mTOR-interacting protein
E6AP	E6-associated protein
EP300	E1A Binding Protein P300
ERK	extracellular signal-regulated kinase
FASN	fatty acid synthase
FBXW7	F-box and WD repeat domain containing 7
FDA	Food and Drug Administration
FLCN	folliculin
FNIP	folliculin interacting protein
GATOR	GTPase activating protein (GAP) activity toward Rags
Grb10	growth factor receptor-bound protein 10
GSK3	glycogen synthase kinase 3
HBXIP	hepatitis B virus X-interacting protein
IAPs	inhibitors of apoptosis proteins
IKKβ	IkappaB kinase beta
IRFs	interferon regulatory factors
JNKs	c-Jun N-terminal protein kinases
KICSTOR	KPTN, ITFG2, C12orf66 and SZT2-containing regulator of mTORC1
KLHL22	Kelch-like protein 22
LATS	Large tumor suppressor kinase
MAPK	mitogen-activated protein kinase
MCL1	myeloid cell leukemia 1
MEFs	mouse embryonic fibroblasts
MiT-TFE	microphthalmia/transcription factor E
mLST8	mammalian lethal with SEC13 protein 8
MTHFD2	methylenetetrahydrofolate dehydrogenase 2
NEDD4L	neural precursor cell expressed developmentally downregulated gene 4-like
NPRL2	Nitrogen permease regulator 2-like protein
NPRL3	Nitrogen permease regulator 3-like protein
NSCLC	Non-small-cell lung carcinoma
O-GlcNAc	O-Linked β-N-acetylglucosamine
OGT	O-linked N-acetylglucosamine transferase
OTUD7B	ovarian tumor domain-containing protein 7B
Pam	protein associated with Myc
PCAF	P300/CBP-associated factor
PDK1	phosphoinositide dependent protein Kkinase 1
PI3K	Phosphoinositide 3-kinase
PKA	protein kinase A
PKC	Protein kinase C
PKM2	pyruvate kinase M2
PPARγ	peroxisome proliferator-activated receptor-gamma
PRAK	p38 regulated/activated kinase
PRAS40	proline-rich Akt substrate of 40 kDa
PTEN	phosphatase and tensin homolog
Rac1	Ras-related C3 botulinum toxin substrate 1
Rap1	Ras-related protein 1
Raptor	regulatory-associated protein of mTOR
Rheb	Ras homolog enriched in brain
PKB	Protein kinase B
Rictor	rapamycin insensitive companion of mTOR
RNF114	ring finger protein 114
RNF152	ring Finger Protein 152
RNF186	ring finger protein 186
RSKs	90 kDa ribosomal S6 kinases
S6K	ribosomal protein S6 kinase
SAMTOR	S-adenosylmethionine sensor upstream of mTORC1
SGK1	serum and glucocorticoid-regulated kinase 1
Sin1	SAPK interacting protein 1
Skp2	S-phase kinase associated protein 2
SREBP1/2	transcriptional factors sterol regulatory element binding protein 1/2
TFEB	transcription factor EB
TRAF2	tumor necrosis factor receptor-associated factor 2
TRAF6	tumor necrosis factor receptor-associated factor 6
TRIM31	tripartite motif-containing protein 31
TSC	Tuberous sclerosis complex
UCH-L1	ubiquitin carboxy-terminal hydrolase L1
ULK1	unc-51 like autophagy activating kinase 1
UVRAG	UV radiation resistance-associated gene protein
VHL	Von Hippel-Lindau
WDR24	WD repeat domain 24
WDR59	WD repeat domain 59
